# Optimizing machine learning models for granular NdFeB magnets by very fast simulated annealing

**DOI:** 10.1038/s41598-021-83315-9

**Published:** 2021-02-15

**Authors:** Hyeon-Kyu Park, Jae-Hyeok Lee, Jehyun Lee, Sang-Koog Kim

**Affiliations:** 1 Nanospinics Laboratory, Department of Materials Science and Engineering, National Creative Research Initiative Center for Spin Dynamics and Spin-Wave Devices, Research Institute of Advanced Materials, Seoul National University, Seoul, 151-744 South Korea; 2 Platform Technology Laboratory, Korea Institute of Energy Research, 152 Gajeong-ro, Yuseong-gu, Daejeon, South Korea

**Keywords:** Materials science, Mathematics and computing

## Abstract

The macroscopic properties of permanent magnets and the resultant performance required for real implementations are determined by the magnets’ microscopic features. However, earlier micromagnetic simulations and experimental studies required relatively a lot of work to gain any complete and comprehensive understanding of the relationships between magnets’ macroscopic properties and their microstructures. Here, by means of supervised learning, we predict reliable values of coercivity (*μ*_0_*H*_*c*_) and maximum magnetic energy product (*BH*_max_) of granular NdFeB magnets according to their microstructural attributes (e.g. inter-grain decoupling, average grain size, and misalignment of easy axes) based on numerical datasets obtained from micromagnetic simulations. We conducted several tests of a variety of supervised machine learning (ML) models including kernel ridge regression (KRR), support vector regression (SVR), and artificial neural network (ANN) regression. The hyper-parameters of these models were optimized by a very fast simulated annealing (VFSA) algorithm with an adaptive cooling schedule. In our datasets of randomly generated 1,000 polycrystalline NdFeB cuboids with different microstructural attributes, all of the models yielded similar results in predicting both *μ*_0_*H*_*c*_ and *BH*_max_. Furthermore, some outliers, which deteriorated the normality of residuals in the prediction of *BH*_max_, were detected and further analyzed. Based on all of our results, we can conclude that our ML approach combined with micromagnetic simulations provides a robust framework for optimal design of microstructures for high-performance NdFeB magnets.

## Introduction

Recently, industrial demands for permanent magnets such as NdFeB (or Nd_2_Fe_14_B) are growing due to their applications to high-performance motors used in electric vehicles (EVs). In particular, NdFeB magnets have attracted intense interest in both research and industrial fields owing to their unique properties as a hard-magnetic material, including outstanding maximum magnetic energy product (*BH*_max_), relatively high coercivity, and lower content of precious rare-earth elements per molecular weight than other hard-magnets such as SmCo_5_. Research on NdFeB magnets has progressed rapidly since their discovery in the 1980s^1^; the highest experimentally observed value of *BH*_max_ has reached ~ 56 MGOe, close to the theoretically calculated maximum intrinsic value of 64 MGOe^2,3^. Nevertheless, much of the study thus far has focused on building up the relationships between macroscopic magnetic properties (e.g. coercivity and *BH*_max_) and microstructural features (e.g. the thickness of grain boundaries^4^, average grain size^5,6^, and the degree of misalignment of easy axes of individual grains^7^) based on experimental observations and finite-element micromagnetic simulations.

Meanwhile, machine learning (ML) is a set of computational methodologies that are capable of learning and recognizing patterns and relationships, based on minimization of error (or an optimization of loss function). Recently, ML-based methods have found great success in the prediction of material properties^8^, the discovery of materials^9^, the design of materials^10^, as well as in the striking reduction of computation time of electronic structure calculation^11^. Application of ML to the fields of hard magnets also has been explored in recent years^12–15^. For example, Möller et al*.*^12^ trained a support vector regression (SVR) model to predict the magnetic material properties of doped NdFeB with less rare-earth contents by combining the ML method with density functional theory. Their model was able to predict the material’s intrinsic magnetic properties, including the saturation magnetization, the anisotropy coefficient, and the Fermi energy, based on given atomic structures with a Pearson correlation coefficient up to 0.92. Meanwhile, Exl et al*.*^13^ utilized a random forest (RF) model in order to characterize the role of microstructural features (e.g. position/size/shape of grains, misalignment of easy axes, etc.) in the switching of an exemplary permanent magnet. The model was able to provide qualitative and quantitative information on which microstructural feature plays the major/minor role in switching. Gusenbauer et al.^14^ used an ensemble method combining RF and gradient boosted regression (GBR) models in order to predict the nucleation field from electron backscatter diffraction (EBSD) images of the surfaces of hard-magnetic MnAl material. They recommended taking advantage of micromagnetic simulation to see the overall trends in the distribution of nucleation fields or to find weak spots in the microstructure. Further, Cheng^15^ employed an SVR model with hyper-parameters obtained by metaheuristic particle swarm optimization in order to correlate, based on experimental data, the chemical composition of materials with their macroscopic magnetic properties such as magnetic remanence, coercivity and *BH*_max_.

However, direct application of ML for prediction of such macroscopic magnetic properties with chemical compositions involves some risks. In general, the coercivities of polycrystalline NdFeB magnets are heavily dependent on microstructural factors as described by the phenomenological relation proposed by Kronmüller and Fähnle^5,6^. Furthermore, inter-grain decoupling is crucial to determination of the switching mechanism, whether it is Stoner-Wohlfarth-type coherent rotation^16^ or Kondorsky-type domain-wall motion^17^. Such different switching mechanisms have been thought to directly impact coercivities^18–20^. Decoupling between individual grains is achieved by spacing out the grains by more than the intrinsic exchange length of bulk NdFeB (~ 1.7 nm), as realized by doping a trace amount of gallium^4^. Thus, the potential of ML to accurately predict the macroscopic properties of NdFeB by employing microstructural attributes needs to be further explored.

In addition, ML models of high accuracy and, at the same time, good quality (i.e. high normality of residual distributions) are desired. Accuracy is determined by a set of mathematical parameters of ML models, called hyper-parameters. Conventionally, hyper-parameters are optimized by brute-force techniques such as grid search^21,22^ and random search^23^, which, however, demand laborious try-and-error procedures and are easily trapped into local minima. Alternatively, simulated annealing is a metaheuristic method that is easy to understand and provides solutions to myriads of optimization problems^24,25^. Like randomized local searching, simulated annealing solves optimization problems by randomly moving from one candidate solution to a neighboring solution, but with a certain probability that depends on differences in energy and current temperature, the latter of which is defined by a cooling schedule. Moreover, good quality of models can be assured by analyzing residuals and quantifying the linearity of their quantile–quantile plots.

**Figure 1:**
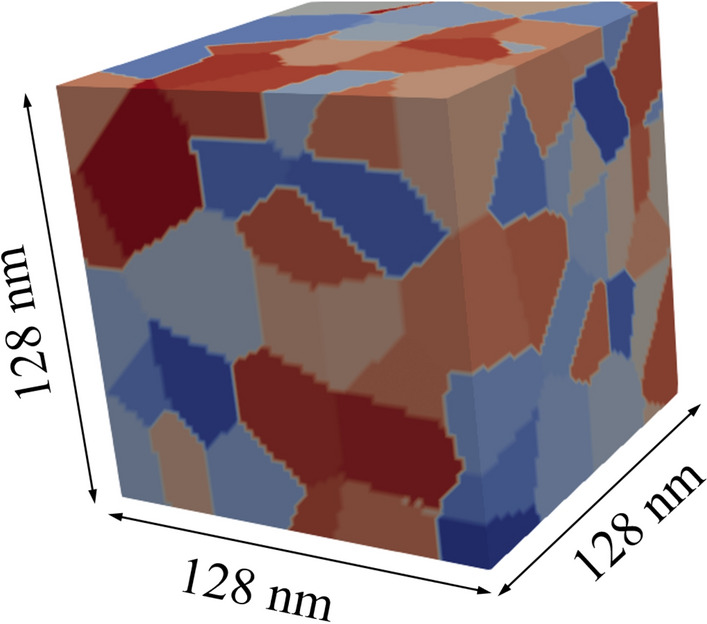
Exemplary polycrystalline NdFeB cuboid of 25.9 nm average grain size with grains indicated by different colors. This figure was created with the open source software ParaView (http://www.paraview.org), licensed under a Creative Commons Attribution 4.0 License.

In this work, we established a database of 1000 different microstructures of polycrystalline NdFeBs (see Fig. 1) of $128{\text{ nm}} \times {\text{128 nm}} \times {\text{128 nm}}$ cuboid geometry using a GPU-accelerated micromagnetic simulation package. We predicted the macroscopic magnetic properties of coercivity and *BH*_max_ by ML models according to microstructural parameters such as inter-grain exchange stiffness *A*_int_, average grain size *D*_grain_, and the degree of misalignment of easy axes of grains *σ*_*θ*_. Moreover, we tested a variety of ML models such as kernel ridge regression (KRR), SVR^26,27^, and artificial neural network (ANN)^28^ with their hyper-parameters optimized by a very fast simulated annealing (VFSA) algorithm that adopts an adaptive cooling schedule. Further, we performed a residual analysis in order to assure the quality of the models, and we detected some outliers that deteriorate model quality in the case of *BH*_max_ prediction. Our results demonstrate the potential of ML methods for future design of NdFeB magnet microstructures in cases where the underlying microstructure-property relationships are not yet clarified.

## Results

### Results of micromagnetic simulations

**Figure 2:**
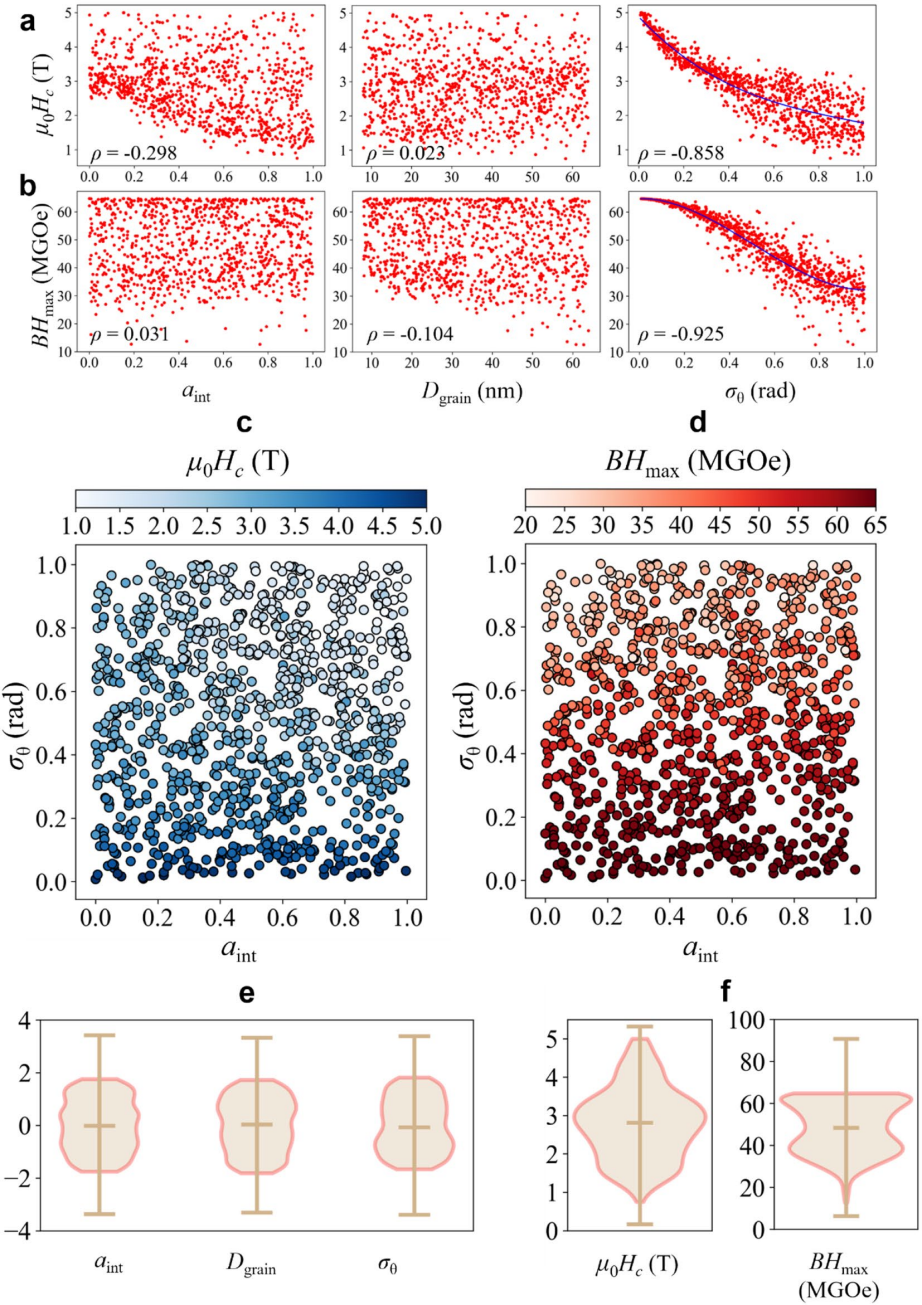
Results of micromagnetic simulations. Scatter plots display the dependence of *a*_int_, *D*_grain_, and *σ*_*θ*_ on **(a)** coercivity and **(b)** *BH*_max_ with the Pearson correlation coefficient (*ρ*) of each plot indicated in the inset. The expressions for polynomial fits are $\mu_{0} H_{c} ({\text{T}}) = - 2.101\sigma_{\theta }^{3} + 5.940\sigma_{\theta }^{2} - 6.942\sigma_{\theta } + 4.875$ and $BH_{\max } ({\text{MGOe}}) = - 66.05\sigma_{\theta }^{3} - 99.98\sigma_{\theta }^{2} + 1.381\sigma_{\theta } + 64.78$. In order to investigate the non-linear correlations between those variables on both coercivity and *BH*_max_, scatter plots are displayed with the *x*-axis indicating *a*_int_, the *y*-axis indicating *σ*_*θ*_, and the color of dots indicating **(c)** coercivity and **(d)** *BH*_max_. Also, violin plots for **(e)** *z*-scores of *a*_int_, *D*_grain_, and *σ*_*θ*_, and **(f)** coercivity and *BH*_max_ are displayed where the outer curves represent the kernel density with the middle line indicating the median. Vertical lines extend and end up in the whiskers, which indicate the lowest and the highest non-outlier data.

In Fig. 2a,b, the dependences of coercivity (*μ*_0_*H*_c_) and *BH*_max_ on reduced parameter *a*_int_ ($= A_{{\text{int}}} /A_{{{\text{ex}}}}$, where *A*_ex_ is the exchange stiffness constant), *D*_grain_, and *σ*_*θ*_ are displayed with the corresponding Pearson correlation coefficient (*ρ*), respectively. Both coercivity and *BH*_max_ increase as *σ*_*θ*_ decreases with the Pearson correlation coefficients of $- 0.858$ and $- 0.925$. Furthermore, both coercivity and *BH*_max_ had a curvilinear relationship that fits with a third-order polynomial formula with respect to *σ*_*θ*_. This resulted from nucleation of reversed domains at higher field strengths and a faster grain-by-grain reversal propagation at higher degrees of alignment of easy axes (i.e. smaller *σ*_*θ*_), as explained in Ref. 7. On the other hand, the dependence of *a*_int_ ($\rho = - 0.298,{ 0}{\text{.031}}$) and *D*_grain_ ($\rho = 0.023, \, - {0}{\text{.104}}$) on either coercivity or *BH*_max_ was observed to be rather weak.

The weak dependence of coercivity on *D*_grain_ can be attributed to the following reason. The dimensions of the grains considered in this work were only 8–64 nm, which are just a few multiples of the exchange length of NdFeB. In such conditions, the coercivity is affected dominantly by the effective magnetic anisotropy rather than the grain-size-dependent demagnetizing factor. In general, when the grain size is larger than a certain critical size (20 nm^5,6^), the coercivity decreases with increasing *D*_grain_, owing to the dominant demagnetization fields, while for grain sizes less than the critical size, the coercivity decreases with decreasing *D*_grain_ owing to the following effective magnetic anisotropy^29^ due, in turn, to the presence of surface defects and imperfection of crystallinity as well as the reduced volume of particles.

On the other hand, in our results, *a*_int_ clearly showed a nonlinear effect on coercivity. In Fig. 2c,d, the distributions of *a*_int_ and *σ*_*θ*_ are scatter-plotted with colors indicating the coercivities and *BH*_max_, respectively. At low *σ*_*θ*_ (i.e. high degree of alignment of easy axes), *a*_int_ has no effect on either coercivity or *BH*_max_. However, at high *σ*_*θ*_ (i.e. low degree of alignment of easy axes), high *a*_int_ turns out to reduce coercivity. However, the same phenomenon was not seen in the *BH*_max_ case, as the Pearson correlation coefficient of 0.031 between *a*_int_ and *BH*_max_ implied. It was revealed that both *a*_int_ and *D*_grain_ were independent of *BH*_max_ in the given *D*_grain_ range of 8–64 nm. Theoretically, for granular magnets of well-aligned easy axes, *BH*_max_ depends only on the remanence squared, provided that the coercivity is greater than $M_{r} /2$, where $M_{r}$ is the remanence^12,30^. Indeed, in our datasets, the remanence showed a strong correlation with the misalignment of easy axes, as shown in Supplementary Information Sect. I. Although there is not much experimental evidence elucidating the relationships between *BH*_max_ and microstructural attributes, a pioneering study of NdFeB^31^ demonstrated that a low *σ*_*θ*_ leads to a high *BH*_max_.

In addition, in order to detect any statistical outliers, we drew violin plots for all of the input/output variables showing the distribution of quartiles for each variable (Fig. 2e,f). Also, we made use of the *z*-scores of input variables, *a*_int_, *D*_grain_, and *σ*_*θ*_ , to visualize the violin plots in the same range of $( - 4, \, 4)$. Consequently, there were no statistical outliers for the input variables or output variables of coercivity and *BH*_max_. In particular, the violin plots for the input variables were nearly symmetric, as they had been sampled from a uniform random distribution. However, the violin plot for *BH*_max_ was biased upward, implying that *BH*_max_ has a “truncated distribution,” because there is a theoretical upper limit for *BH*_max_ that is 64 MGOe^3^.

### Sampling of training and test datasets

As discussed in this section, we trained KRR, SVR, and ANN models using 1000 examples of coercivity and *BH*_max_ calculated from each polycrystalline sample with different *a*_int_, *σ*_*θ*_, and *D*_grain_. The 1000 pairs of datasets were split into 800 training sets and 200 test sets, and the training sets were further sub-divided into 600 training and 200 validation sets for optimization by the VFSA algorithm, using root-mean-squared errors (RMSE). We normalized each input data for different *a*_int_, *σ*_*θ*_, and *D*_grain_ by making use of the *z*-score of each input data,

$$ z = \frac{x - \mu }{\sigma }, $$

(*x*: input data, *μ*: mean, *σ*: standard deviation) so as to have a distribution $\sim \user2{\mathcal{N}}(0, \, 1)$. This procedure enhances the performance of ML models^32^. Also, we utilized the python packages of the scikit-learn implementations for each model, and made use of a VFSA metaheuristics algorithm in order to optimize the typical hyper-parameters concerned with each model. Using the sampled data, we optimized each KRR, SVR, and ANN models by employing the VFSA algorithm and an adaptive cooling schedule.

### Training of models by VFSA

**Figure 3:**
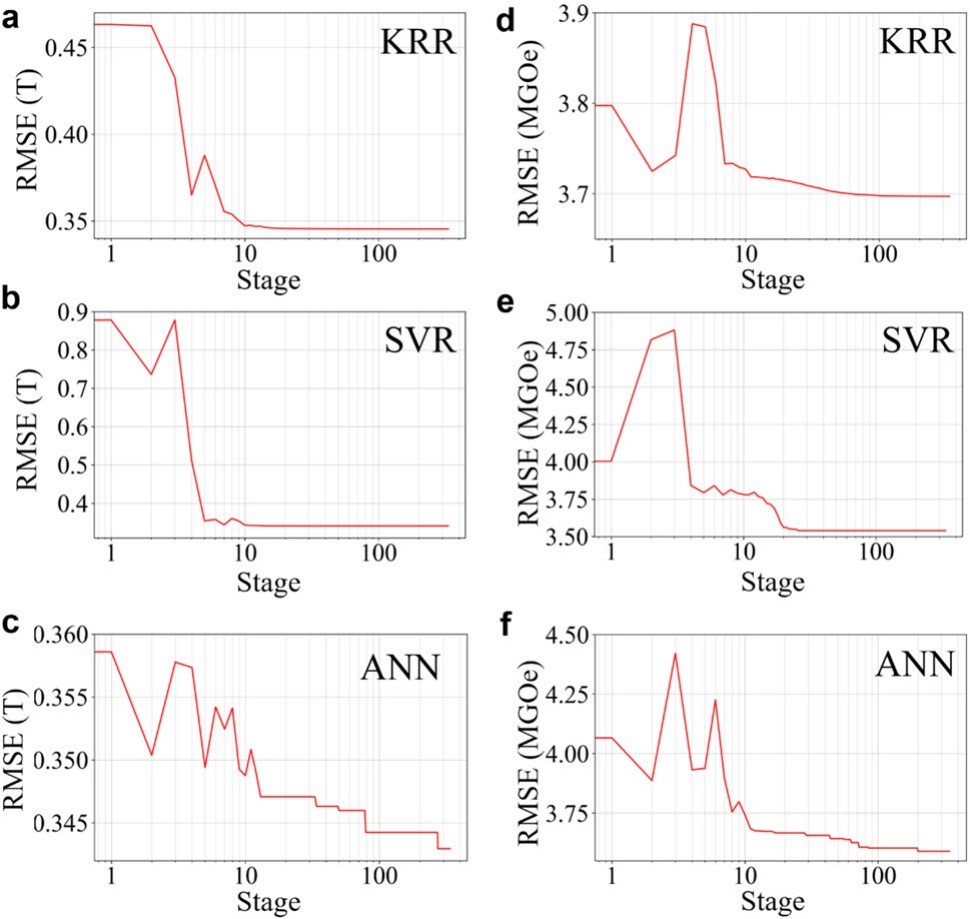
Profiles of RMSE by VFSA coupled with different ML models. The profile of the RMSE between the predicted and actual values of coercivity as optimized with the **(a)** KRR, **(b)** SVR, and **(c)** ANN models and of *BH*_max_ as optimized with the **(d)** KRR, **(e)** SVR, and **(f)** ANN models indicate two phases: a high degree of randomness at the initial stages (1–10) and a gradual minimization at the latter stages (10–100).

In Fig. 3a–f, the profiles of RMSE versus all of the stages are displayed for optimization of coercivity prediction (Fig. 3a–c) and of *BH*_max_ prediction (Fig. 3d–f) for each model. At the initial stages of the RMSE profile, a high degree of randomness was maintained for the initial stages (1–10), where the candidate solution escaped from the local minima of the objective function landscape. Nonetheless, in the latter stages (10–100), all of the RMSEs were well minimized via simulated annealing, essentially quenched into the global minimum of the energy landscape. The values of hyper-parameters obtained via VFSA are summarized in Table 1.

**Table 1:** Hyper-parameter values obtained by VFSA for three ML models for the prediction for both coercivity and *BH*_max_.

Model	Hyper-parameters	Values for coercivity prediction	Values for *BH*_max_ prediction
KRR	*α*	1.7342 × 10^–6^	2.6823 × 10^–10^
	*γ*	7.7116 × 10^–4^	7.3982 × 10^–3^
SVR	*C*	43.598	1.6339
	*γ*	0.27035	0.11523
	*ε*	3.3378 × 10^–2^	1.5773
ANN	*α*	5.4887 × 10^–3^	0.17893
	Activation function type	tanh	ReLU

### Prediction by various ML models

**Figure 4:**
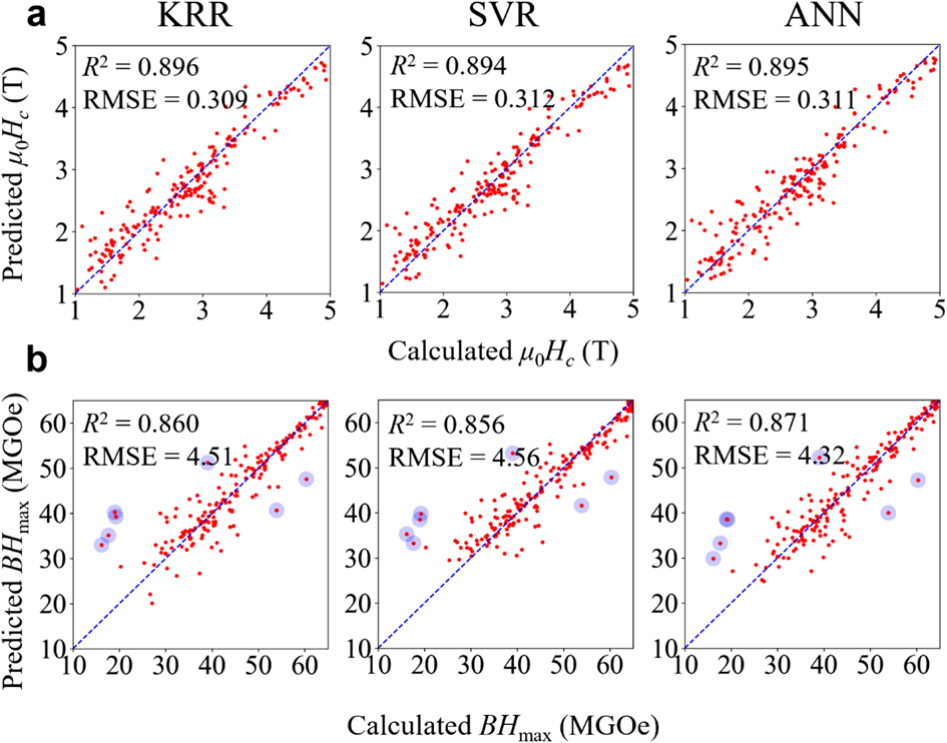
Prediction of final ML models optimized by VFSA with coefficient of determination (*R*^2^) and RMSE indicated as insets. **(a)** Prediction of coercivity by KRR, SVR, and ANN models. **(b)** Prediction of *BH*_max_ by KRR, SVR, and ANN models. The units of RMSE in **(a)** and **(b)** are T and MGOe, respectively. The blue translucent dots in **(b)** indicate the seven outliers with the largest residuals.

Now, we are ready to present the main findings of this work, which is the prediction of coercivity and *BH*_max_ by various the three ML models (i.e. KRR, SVR, and ANN) optimized by VFSA. Our goal was to choose and make use of the most appropriate ML method to approximate the implicit relationships between the microstructural attributes of *a*_int_, *D*_grain_, and *σ*_*θ*_ and the macroscopic magnetic properties of coercivity and *BH*_max_. Figure 4a,b show, respectively, the prediction of coercivity and *BH*_max_ for the unseen test pairs using the KRR, SVR, and ANN models. The coefficient of determination (*R*^2^) and RMSE of the coercivity and *BH*_max_ for the test cases are summarized therein. For parity plots of the training datasets, see Supplementary Information Sect. II. The reasonable agreement between the ML prediction and micromagnetics calculation shows the predictive ability of the models even when using only a handful of microstructural features.

### Residual analysis

**Figure 5:**
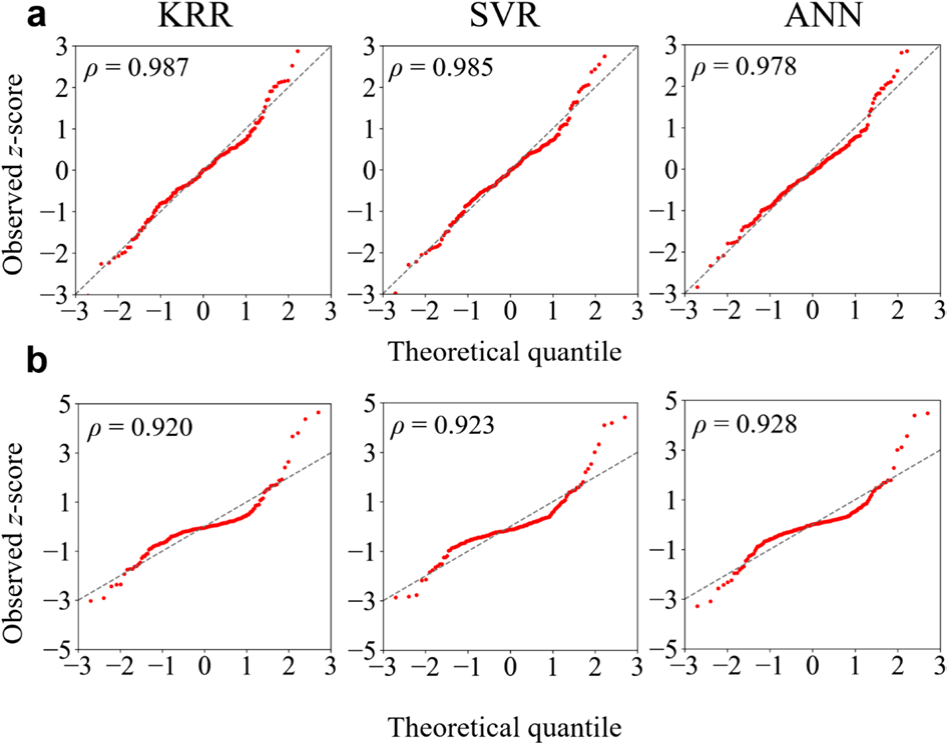
Q–Q plots for *z*-scores of residuals between predicted and actual values with Pearson correlation coefficient of each plot indicated in insets. **(a)** Q–Q plots obtained from prediction of coercivity by KRR, SVR, and ANN models. **(b)** Q–Q plots obtained from prediction of *BH*_max_ by KRR, SVR, and ANN models. The gray lines in each graph are the trend lines to which the ideal Q–Q plot in each case should correspond.

Furthermore, in the prediction results for *BH*_max_, we identified seven outliers (blue translucent dots in Fig. 4b) that had the largest biases between the prediction and real data value. We found out that, by the presence of these outliers, the normality of residuals for the ML models predicting *BH*_max_ was broken. In Fig. 5a, b, quantile–quantile (Q–Q) plots for the residuals between the predictions and real datasets are displayed. Note that an unbiased model would have a normal distribution of residuals and thus a linear Q–Q plot. Then, we again normalized the residuals in order to compare them with a normal distribution and plotted them against the theoretical quantiles of the normal distribution. In terms of the Pearson correlation coefficient, the Q–Q plots were almost linear ($\rho \approx 1$) in the cases of the coercivity predictions of the three ML models, whereas they were non-linear ($\rho \ll 1$) in the cases of *BH*_max_. Nonetheless, we found that over-fitting, as indicated by four-fold cross-validation, was not detected, as shown in Supplementary Information Sect. III.

## Discussion

**Figure 6:**
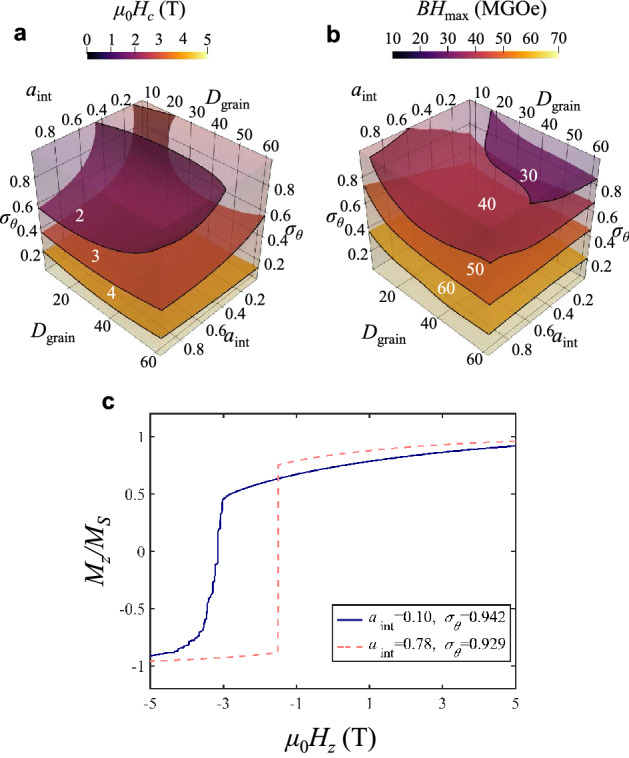
Dependences of coercivity and *BH*_max_ on input parameters. Ensemble of **(a)** coercivities and **(b)** *BH*_max_ values predicted from KRR, SVR and ANN models. The annotated numbers on the contour planes denote the values of coercivity and *BH*_max_ in the units of T and MGOe, respectively. The contour plots were created with ParaView. **(c)** Demagnetization curves for different sets of indicated *a*_int_ and *σ*_*θ*_ values.

In order to overview the dependences of coercivity and *BH*_max_ with respect to the input parameters, we predicted those values from 42,875 artificially generated data as shown in Fig. 6a,b. The predictions were obtained from ensemble averages of coercivity and *BH*_max_ from the KRR, SVR, and ANN models, as shown in Supplementary Information Sect. IV. The three-dimensional plots revealed the dominance of the three different input parameters (i.e., the misalignment of easy axes of grains, inter-grain exchange coupling, and grain size) in determining coercivity and *BH*_max_. Note that for sufficiently large misalignments of the easy axes, the dependences of coercivity and *BH*_max_ on inter-grain exchange coupling are opposite to each other. The weak inter-grain exchange coupling slightly lowers remanent magnetization and the overall coercivity, but also prevents the propagation of the reversed domains into the neighboring grains, which makes the nucleation-controlled magnetization reversal process more preferable^33^. In Fig. 6c are shown two different demagnetization curves representing weak and strong inter-grain exchange coupling (*a*_int_ = 0.10 vs. 0.78) for sufficiently large misalignments (*σ*_*θ*_ = 0.942 and 0.929).

**Figure 7:**
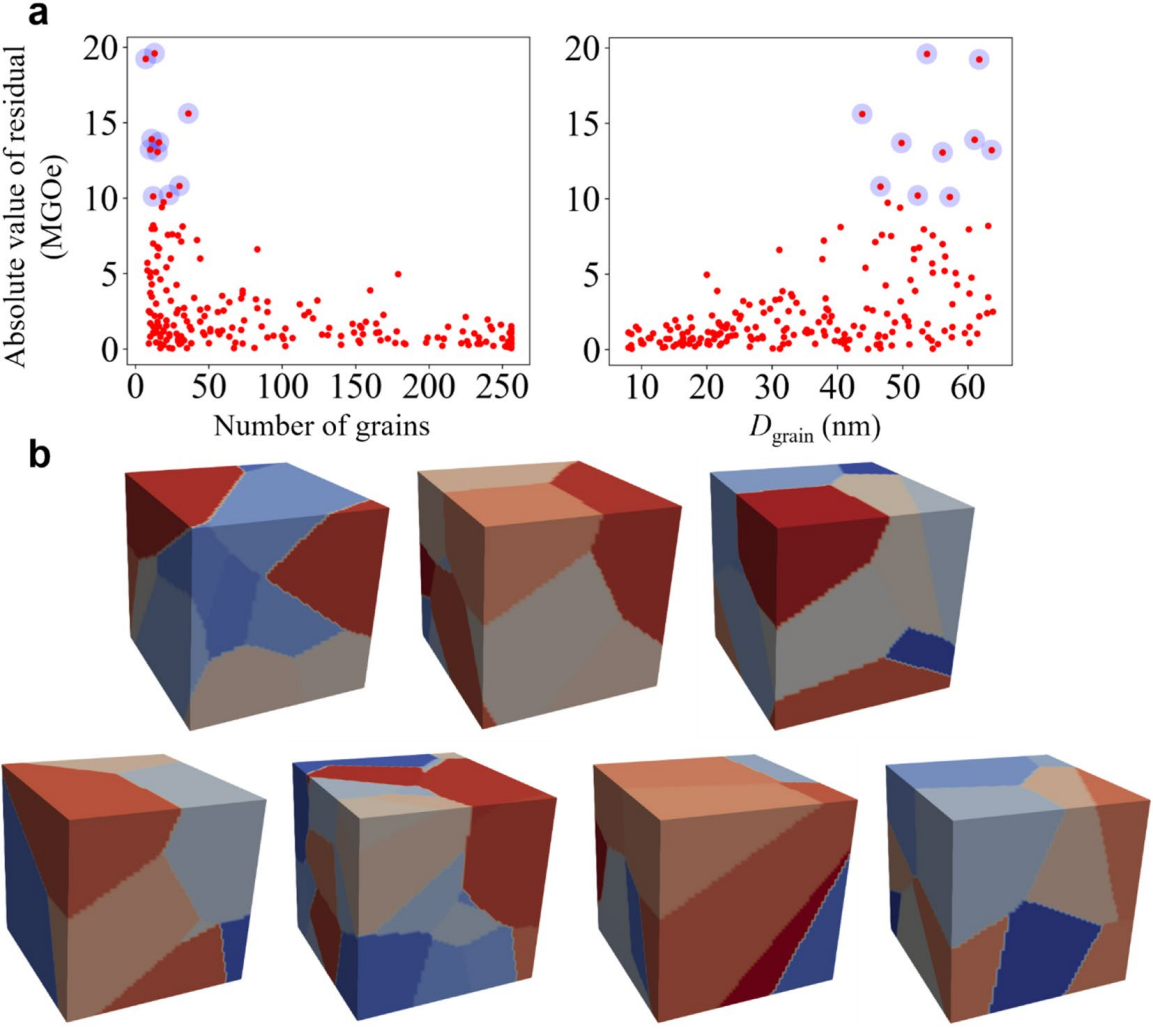
Origin of seven outliers. **(a)** Residuals between predicted and actual values of *BH*_max_ against the number of grains and *D*_grain_ as obtained using ANN model. The seven outliers with residuals larger than 13 MGOe are indicated by the blue translucent dots. **(b)** Model cuboids for seven outliers with grains indicated by different colors. The model cuboids were visualized with ParaView.

A few data were detected as outliers, particularly in the *BH*_max_ prediction, as marked with the blue dots in Fig. 4b, because there were unusual features involved in their corresponding model geometry. As explained in Ref.^13^, the weakest grains in a polycrystalline hard-magnetic cuboid are placed at the edges of the upside or downside plane of cuboids because demagnetization fields are concentrated there. That is, whether a grain is weak or not is largely determined by its geometrical position inside of cuboids. As the number of grains per cuboid decreases, both the average size of grains and the surface-to-volume ratio of each grain increase. Thus, the portion of weakest grains, which cover the surfaces, is higher in a coarse-grained cuboid than in a fine-grained one. Figure 7a demonstrates in the case of the ANN model, where the seven outliers were all found in coarse-grained cuboids, or cuboids with large *D*_grain_ or a small number of grains. Also, Fig. 7b displays the cuboid models for each of the seven outliers, where large and coarse grains occupy the surfaces of the cuboid. We believe that over- or under-estimation of predicted values of *BH*_max_ occurred in those specific coarse-grained cuboids, because the ML models were unable to consider the irregular changes of *BH*_max_ in them. Regardless, further studies are needed for a more qualitative description.

We expect that GPU-based micromagnetic simulations and optimization of ML models by metaheuristics such as simulated annealing, genetic algorithm, and tabu search would facilitate the optimal design and/or process of microstructures of hard magnets with the aid of advanced fabrication technologies. For example, minutely increased grain boundary width and external alignment field leads to substantial decoupling between grains^4^ and grain alignment^31^. Furthermore, in the past, ML models poorly optimized by brute-force techniques such as grid search and random search were adopted in a variety of studies^21–23^. The metaheuristics we employed in this study, VFSA, are based on a concept easy to understand and employ. As such, our work can be said to provide a cornerstone for future ML studies employing VFSA.

In summary, in order to predict the coercivity and *BH*_max_ of NdFeB magnets by ML and search for appropriate models, first we constructed, by micromagnetic simulations, a dataset of the correlation between the microstructural features of granular NdFeB magnets (average grain size, misalignment of easy axes, inter-grain decoupling) and their macroscopic properties (coercivity and *BH*_max_). We revealed that ML models combined with VFSA and an adaptive cooling schedule well predict, according to a variety of microstructural parameters, the coercivity as well as *BH*_max_ of NdFeB magnets. Coercivity had little relationships with respect to *D*_grain_ but had a non-linear type of relationship with respect to both *a*_int_ and *σ*_*θ*_. This unusual behavior contradicts the phenomenological theory whereby coercivities are linearly dependent on grain sizes on ~ *μ*m scales. We believe that this partly results from the averaged-out irregular shape factors. On the other hand, *BH*_max_ had a non-linear type of relationship with respect only to misalignment of easy axes. These results, though obtained under the specific conditions of grain sizes on ~ nm scales, are invaluable in that only a few researchers^31^ have experimentally attempted to correlate *BH*_max_ with microstructural factors. Based on the present application of the VFSA method combined with the KRR, SVR, and ANN models, it was determined that all of the models provided similar performances in predicting both coercivity and *BH*_max_. Especially, for the prediction of *BH*_max_, we detected seven outliers (i.e. over- or under-estimation of *BH*_max_) due to which the quality of the used models was deteriorated. These outliers had appeared owing to too-large sizes of grains covering the top and/or bottom of the cuboid geometry, leading to irregular values of *BH*_max_ that the models could not consider. Further, the elimination of those outliers resulted in much better performance in the prediction of *BH*_max_, yielding better-quality ML models. The ML combined with micromagnetic simulation study provided a robust framework for the design of optimal microstructures of high-performance NdFeB magnets without any need for painstaking micromagnetic simulations and/or delicate experiments. Furthermore, our results demonstrated the potential of ML for the design of optimal microstructures of NdFeB magnets, notwithstanding the fact that the underlying microstructure-property relationships remain unclear.

## Methods

### Micromagnetic simulations

For reliable learning of training data, a large number of datasets including demagnetization and *B*–*H* curves should be prepared. For this purpose, we employed a GPU-accelerated micromagnetics package, Mumax3, which incorporates the Landau-Lifshitz-Gilbert (LLG) equation. The package, based on a finite difference method, calculates the demagnetization curves for a single polycrystalline NdFeB system composed of $64 \times 64 \times 64$ cells. We used the ‘ext_make3dgrain’ function incorporated into Mumax3 in order to generate the polycrystalline granular structures. Since this function is based on three-dimensional Voronoi tessellation with randomly chosen crystal seeds, the distribution of grain sizes in our multi-grain model was totally random. We generated all of the necessary codes responsible for 1000 polycrystalline NdFeB models, and executed each code in order to obtain the demagnetization curve and the corresponding *B*–*H* curve, from which coercivity and *BH*_max_ were extracted, respectively.

Each simulation model had $5 - 256$ grains with average grain sizes (*D*_grain_) ranging from 8 to 64 nm. Further, in order to examine the effect of misalignment of individual grains’ uniaxial magnetic anisotropy orientation on coercivity and *BH*_max_, we assumed Gaussian distributions^34^ with standard deviations of $\sigma_{\theta } ({\text{rad}}) \in [0,{ 1]}$ for the angle between the grains’ easy axis and *z*-axis, *θ*. Here, the bound of 1 rad corresponds to the average alignment of easy axes when the perpendicular aligning field is 0.05 T ^31^. We utilized the following magnetic parameters corresponding to NdFeB^35^: saturation magnetic polarization $J_{S} = 1.61{\text{ T}}$, exchange stiffness constant $A_{{{\text{ex}}}} = 12.5{\text{ pJ/m}}$, reduced parameter $a_{{\text{int}}} = A_{{\text{int}}} /A_{{{\text{ex}}}} \in [0, \, 1]$ where $A_{{\text{int}}}$ is the inter-grain exchange stiffness constant, and first-order magnetic anisotropy constant $K_{1} = 4.5{\text{ MJ/m}}^{3}$. The size of mesh discretizing the cuboid model was set to $2{\text{ nm}}$, which is close to the exchange length of NdFeB material, $\sqrt {{{A_{{{\text{ex}}}} } \mathord{\left/ {\vphantom {{A_{{{\text{ex}}}} } {K_{1} }}} \right. \kern-\nulldelimiterspace} {K_{1} }}} = 1.7{\text{ nm}}$.

### Details of ML models

The microstructural features used to train the models were of three types: reduced inter-grain exchange stiffness (*a*_int_), average grain size (*D*_grain_), and degree of misalignment of easy axes (*σ*_*θ*_). In the present work, all of the optimization problems were solved by scikit-learn implementation. The hyper-parameters of each supervised ML models were optimized by the VFSA algorithm^36,37^. The types and details of the ML models employed in this work are as follows.

#### Kernel ridge regression

Kernel ridge regression (KRR) is a classic approach that constrains model parameter magnitudes. It limits the sum of squared errors by imposing an *L*_2_-norm, which is the sum of squares of weights **w**. Given a training dataset $\left\{ {\left( {{\mathbf{x}}_{1} , \, y_{1} } \right), \, \cdots \, ,\left( {{\mathbf{x}}_{n} , \, y_{n} } \right)} \right\}$, this is equivalent to minimizing the objective function^38^

$$ \frac{1}{2}\sum\limits_{i = 1}^{n} {(y_{i} - {\mathbf{w}}^{T} \phi ({\mathbf{x}}_{i} ))^{2} } + \frac{1}{2}\alpha \left\| {\mathbf{w}} \right\|^{2} $$

where $\phi :{\mathbb{R}}^{n} \to {\mathbb{R}}$ is a kernel function that maps ${\mathbf{x}}_{i} \in {\mathbb{R}}^{n}$ to the feature space. In this work, a radial basis function $\phi ({\mathbf{x}}_{i} ) = \exp ( - \gamma \left\| {{\mathbf{x}}_{i} } \right\|^{2} )$ was employed as the kernel function. The second term is the regularization term in which *α* acts as a weight that balances minimization of the sum of squared errors and limits the complexity of the model. In general, the larger the value of *α*, the lower the magnitude of parameters and thus of the complexity of the model^38^. There were two hyper-parameters of KRR model to be optimized: the coefficient of the kernel function $\gamma$ and the regularization parameter $\alpha$.

#### Support vector regression

Support vector regression (SVR) is a non-linear regression analysis based on support vector machine, which is again rooted in statistical learning or Vapnik–Chervonenkis theory^26,27^. The loss functions for ordinary regression analysis are sums of squares of error, whereas that of SVR is an *ε*-insensitive loss function of linear, quadratic, or Huber type. In *ε*-SVR, the goal is to find a function *f*(**x**) that has at most *ε* deviation from the actually obtained targets *y*_i_ for all training data, and at the same time is as flat as possible, i.e. with as small weights as possible.

Suppose we are given a training dataset $\left\{ {\left( {{\mathbf{x}}_{1} , \, y_{1} } \right), \, \cdots \, ,\left( {{\mathbf{x}}_{n} , \, y_{n} } \right)} \right\}$, where **x**_*i*_ is a vector of independent variables and *y*_*i*_ is a corresponding scalar-dependent variable. Then, the function in the feature space is approximated by $f({\mathbf{x}}) = {\mathbf{w}}^{T} \phi ({\mathbf{x}}) + b$, where **w** defines the weight vector, *b* is a bias parameter, and $\phi ({\mathbf{x}})$ is a kernel function that maps x to the feature space. In the present work, a radial basis function $\phi ({\mathbf{x}}_{i} ) = \exp ( - \gamma \left\| {{\mathbf{x}}_{i} } \right\|^{2} )$ was employed as the kernel. The loss function to be minimized is described by

$$ \frac{1}{2}\left\| {\mathbf{w}} \right\|^{2} + C\sum\limits_{i = 1}^{n} {E_{\varepsilon } (y_{i} ,f({\mathbf{x}}_{i} ))} $$

where *C* is the regularization parameter and $E_{\varepsilon } (y,f({\mathbf{x}}_{i} ))$ is the *ε*-insensitive loss function. There were three hyper-parameters to be optimized: *C*, *ε,* and *γ*.

#### Artificial neural network

For artificial neural network (ANN) regression^28^, we used the MLPRegressor module implemented in the scikit-learn package. The *L*_2_ regularization parameter, *α*, and the type of activation functions (allowed to shift between a hyperbolic tangent function (tanh), a sigmoid function (logistic), and a rectified linear unit function (ReLU)) employed in this method were two hyper-parameters to be optimized. However, the optimization method was restricted to the limited-memory Broyden–Fletcher–Goldfarb–Shanno (L-BFGS) method, as were the number of hidden layers and neurons, to 1 and 100, respectively, for simplicity. In addition, we used the mean squared error with *L*_2_-penalty as the loss function.

### Simulated annealing

We employed the VFSA algorithm proposed by Szu and Hartley^39^ and improved by Ingber^40^. Also, we adopted an adaptive cooling schedule, according to which the temperature at the $j$th stage is calculated by

$$ T_{j + 1} = \frac{{T_{j} }}{{1 + \exp [ - (f({\mathbf{x}}_{{{\text{cand}}}} ) - f({\mathbf{x}}_{{{\text{curr}}}} ))/T_{0} ]}}, $$

where $f({\mathbf{x}})$ is the objective function to optimize, ${\mathbf{x}}_{{{\text{curr}}}}$ is the current solution, ${\mathbf{x}}_{{{\text{cand}}}}$ is the candidate solution, and $T_{0}$ is the initial temperature. This kind of cooling scheme is based on idea that keeps the temperature unchanged when the value of the objective function for the candidate solution is far from that for the global optimum and that halves the temperature when the solution is updated ($f({\mathbf{x}}_{{{\text{curr}}}} ) = f({\mathbf{x}}_{{{\text{cand}}}} )$). The RMSE between the actual datasets as calculated from micromagnetic simulation and those predicted from ML model was used as the objective function in this scheme. Further, the initial temperature was set such that the acceptance probability at the initial stage is 0.7, in order to avoid redundant initial stages with a high degree of randomness^41^, and the final temperature was set to be sufficiently low, at $10^{ - 100}$. At each temperature, the neighborhoods of the candidate solution were searched 100 times.

## Supplementary Information

Supplementary Information.
