# Supplementary material for: Optimizing machine learning models for granular NdFeB magnets by very fast simulated annealing
Source: Sci Rep. 2021 Feb 15;11:3792. doi: 10.1038/s41598-021-83315-9 (PMC7884417; doi:10.1038/s41598-021-83315-9)
Supplement: Supplementary file 1 — Supplementary Information. [file 41598_2021_83315_MOESM1_ESM.pdf]

Supplementary Information for

Optimizing machine learning models for granular NdFeB magnets by very fast simulated annealing

Hyeon-Kyu Park[1], Jae-Hyeok Lee[1], Jehyun Lee[2,a)], and Sang-Koog Kim[1,b)]

[1] *National Creative Research Initiative Center for Spin Dynamics and Spin-Wave Devices, Nanospinics Laboratory, Research Institute of Advanced Materials, Department of Materials Science and Engineering, Seoul National University, Seoul 151-744, South Korea*

[2] *Platform Technology Laboratory, Korea Institute of Energy Research, 152 Gajeong-ro, Yuseong-gu, Daejeon, South Korea*

[a), b)] Authors to whom correspondence should be addressed. Electronic mail: sangkoog@snu.ac.kr

I. Dependence of a_{int}, D_{grain}, σ_θ on remanence

We further examined remanence M_r from the hysteresis curves. Scatter-plots of the dependence of reduced remanence M_r/M_S on each of the input parameters, a_{int}, D_{grain}, and σ_θ are given (see Fig. S1). In terms of Pearson correlation coefficient ρ, a_{int} and D_{grain} showed weak correlations with M_r, as indicated by ρ = 0.042 and -0.119, respectively. On the other hand, the remanence showed the strongest correlation with σ_θ, as manifested by ρ = -0.897. Such a strong dependence of BH_{max} with the misalignment of easy axes of grains agrees well with the fact that BH_{max} for granular magnets of well aligned easy axes depends only on the remanence squared, provided that the coercive field is greater than $M_r/2$.

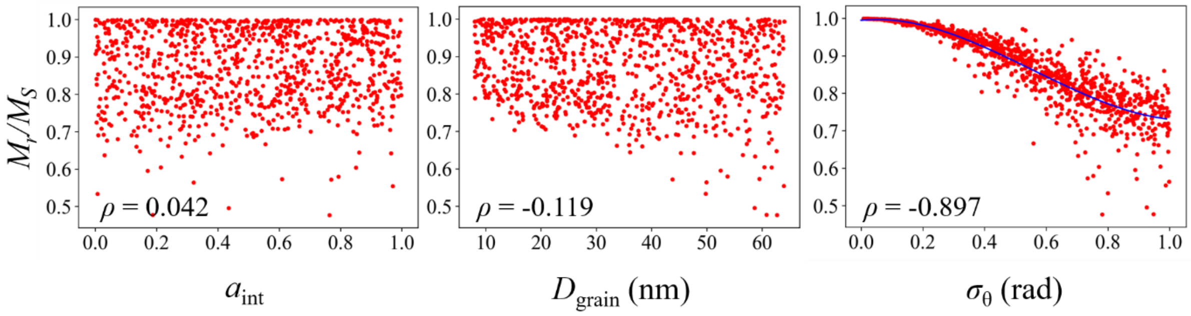

Figure S1 | Scatter plot of reduced remanence (M_r/M_S) as function of each input variable a_{int}, D_{grain}, and σ_θ. Pearson correlation coefficient ρ is provided in the lower-left of each graph. The blue curve on the right indicates the third-order polynomial fit to data using M_r/M_S = $0.995+0.088\sigma_\theta-0.882\sigma_\theta^2+0.530\sigma_\theta^3$.

II. Parity plots for training datasets

Parity plots for the training datasets are shown in Fig. S2. The coefficients of determination R^2 and root mean square error (RMSE) between the training datasets and the predictions are given for each of the KRR, SVR, and ANN models.

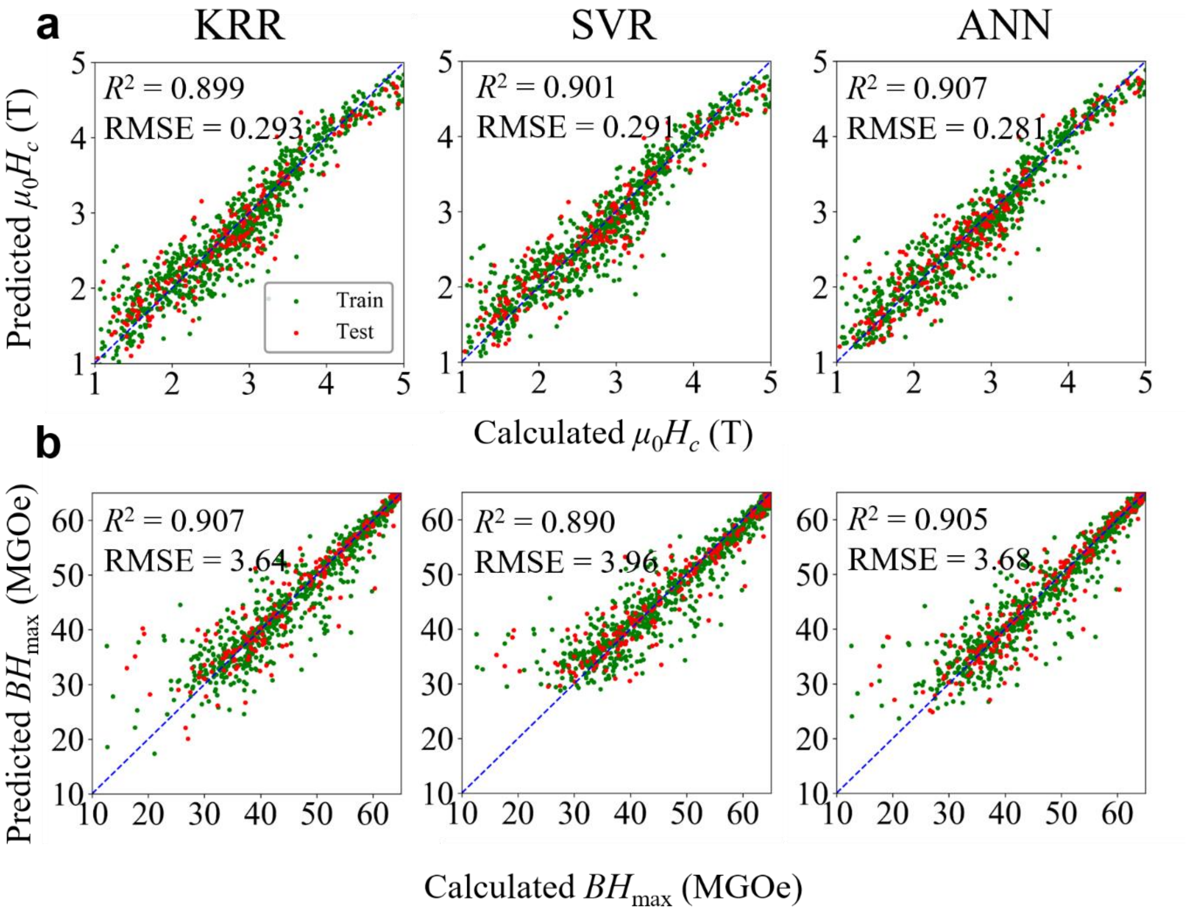

Figure S2 | Parity plots for both training and test datasets for **(a)** coercivity and **(b)** BH_{max} . The green dots indicate the training datasets and the corresponding prediction, while the red dots indicate the test datasets and the corresponding prediction.

III. Cross-validation test for models optimized by VFSA

We conducted cross-validation tests by dividing the training datasets according to four folds in order to determine if each model was over-fitted and/or biased. Tables S1 and S2 summarizes the results of the cross-validation tests for coercivity and BH_{max}, respectively. The mean RMSE values were close to those from the models optimized by VFSA (0.309 ~ 0.312 T and 4.32 ~ 4.56 MGOe). Consequently, overfitting was not detected for either coercivity or BH_{max}. However, for BH_{max}, biases between different folds were present, due to the coarse-grained models.

Table S1 | Cross-validation tests for coercivity optimized by VFSA. The RMSEs of each of the folds given by the four-fold cross-validation tests are summarized.

Model	RMSE for Fold 1 (T)	RMSE for Fold 2 (T)	RMSE for Fold 3 (T)	RMSE for Fold 4 (T)	Mean (Std. dev.) (T)
KRR	0.303	0.326	0.269	0.284	0.295 (0.021)
SVR	0.303	0.330	0.271	0.280	0.296 (0.023)
ANN	0.298	0.319	0.261	0.270	0.287 (0.023)

Table S2 | Cross-validation tests for BH_{max} optimized by VFSA. The RMSEs of each of the folds given by the four-fold cross-validation tests are summarized.

Model	RMSE for Fold 1 (MGOe)	RMSE for Fold 2 (MGOe)	RMSE for Fold 3 (MGOe)	RMSE for Fold 4 (MGOe)	Mean (Std. dev.) (MGOe)
KRR	4.395	4.089	4.311	3.781	4.144 (0.238)
SVR	4.490	4.062	4.105	3.447	4.026 (0.373)
ANN	4.309	4.141	3.989	3.494	3.983 (0.304)

IV. Hyper-planes of each model

We further visualized the prediction results for H_c and BH_{max} according to input parameters a_{int}, D_{grain}, and σ_θ by the KRR, SVR, and ANN models using more than 40,000 artificially generated data (see the contour plots in Fig. S3). The grain-size (D_{grain}) dependences on either coercivity or BH_{max} were negligible relative to those on misalignment or inter-grain exchange coupling. For the purpose of ensuring model reliability, an ensemble model was constructed by averaging the three models.

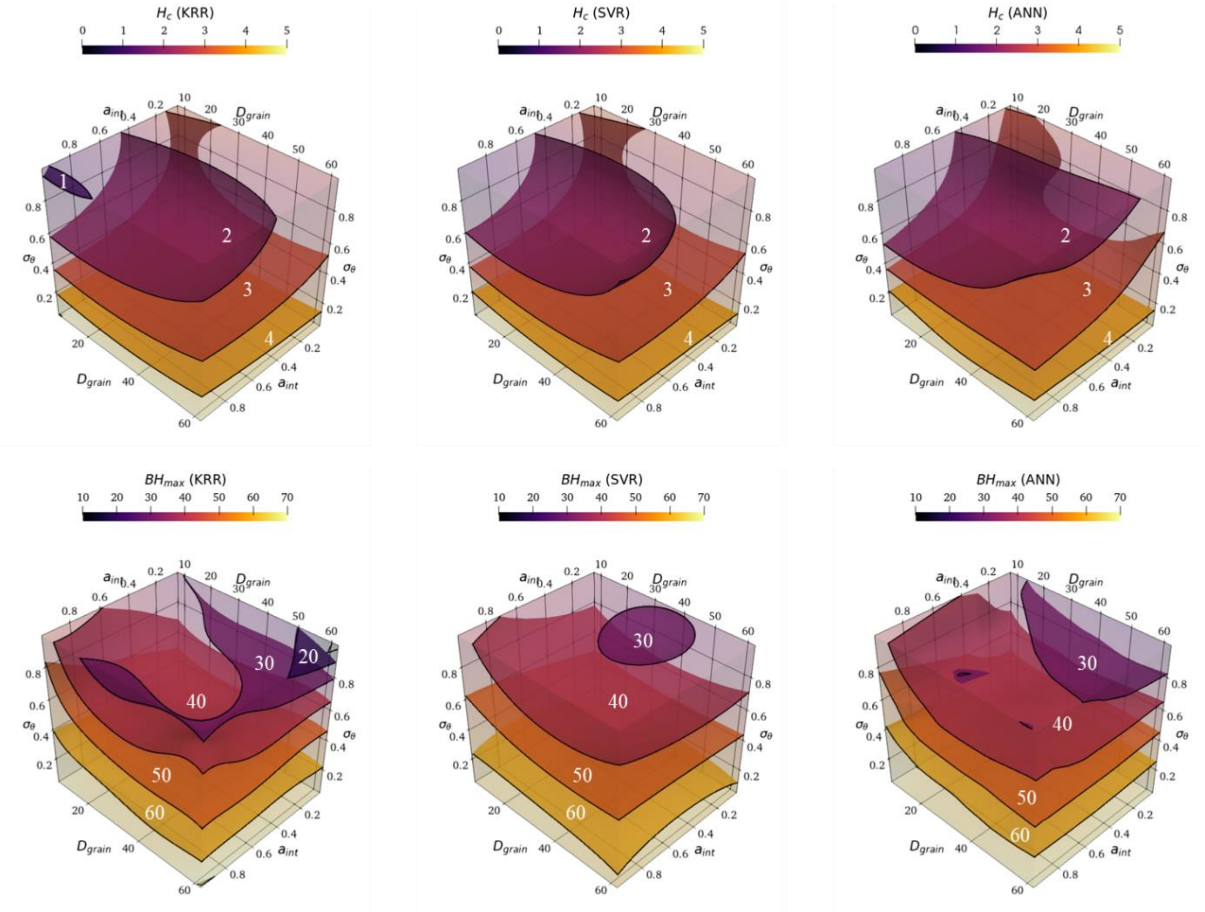

Figure S3 | Dependence of coercivity and BH_{max} on input variables for KRR, SVR and ANN models. The annotated numbers on the contour planes denote the values of coercivity and BH_{max}. The units of coercivity and BH_{max} are T and MGOe, respectively. This figure was created with the open source software ParaView.
